# Surgical Apgar score as a predictor of outcomes in patients following laparotomy at Mulago National Referral Hospital, Uganda: a prospective cohort study

**DOI:** 10.1186/s12893-022-01883-7

**Published:** 2022-12-18

**Authors:** Bruno Chan Onen, Andrew Weil Semulimi, Felix Bongomin, Ronald Olum, Gideon Kurigamba, Ronald Mbiine, Olivia Kituuka

**Affiliations:** 1 Department of Surgery, School of Medicine, College of Health Sciences, Makerere University, P.O. Box 7072, Kampala, Uganda; 2 Lung Institute, Department of Medicine, School of Medicine, College of Health Sciences, Makerere University, Kampala, Uganda; 3 Department of Medical Microbiology and Immunology, Faculty of Medicine, Gulu University, P.O. Box 166, Gulu, Uganda; 4 School of Medicine, College of Health Sciences, Makerere University, P.O. Box 7072, Kampala, Uganda; 5 Department of Surgery, Mulago National Referral Hospital, Kampala, Uganda

**Keywords:** Surgical Apgar score, Laparotomy, Major complications

## Abstract

**Background:**

Postoperative complications and mortality following laparotomy have remained high worldwide. Early postoperative risk stratification is essential to improve outcomes and clinical care. The surgical Apgar score (SAS) is a simple and objective bedside prediction tool that can guide a surgeon’s postoperative decision making. The objective of this study was to evaluate the performance of SAS in predicting outcomes in patients undergoing laparotomy at Mulago hospital.

**Method:**

A prospective observational study was conducted among eligible adult patients undergoing laparotomy at Mulago hospital and followed up for 4 months. We collected data on the patient’s preoperative and intraoperative characteristics. Using the data generated, SAS was calculated, and patients were classified into 3 groups namely: low (8–10), medium (5–7), and high (0–4). Primary outcomes were in-hospital major complications and mortality. Data was presented as proportions or mean (standard deviation) or median (interquartile range) as appropriate. We used inferential statistics to determine the association between the SAS and the primary outcomes while the SAS discriminatory ability was determined from the receiver-operating curve (ROC) analysis.

**Results:**

Of the 151 participants recruited, 103 (68.2%) were male and the mean age was 40.6 ± 15. Overall postoperative in-hospital major complications and mortality rates were 24.2% and 10.6%, respectively. The participants with a high SAS category had an18.4 times risk (95% CI, 1.9–177, p = 0.012) of developing major complications, while those in medium SAS category had 3.9 times risk (95% CI, 1.01–15.26, p = 0.048) of dying. SAS had a fair discriminatory ability for in-hospital major complications and mortality with the area under the curve of 0.75 and 0.77, respectively. The sensitivity and specificity of SAS ≤ 6 for major complications were 60.5% and 81.14% respectively, and for death 54.8% and 81.3%, respectively.

**Conclusion:**

SAS of ≤ 6 is associated with an increased risk of major complications and/or mortality. SAS has a high specificity with an overall fair discriminatory ability of predicting the risk of developing in-hospital major complications and/or death following laparotomy.

## Introduction

Globally, over seven million people develop postoperative complications annually [1], while postoperative deaths are the third leading contributor to global mortality with 7.7% of the mortality occurring within 30 days of surgery [2]. Half of global mortality and complications occur in low-and middle-income countries (LMICs) [3] with 20% of patients undergoing surgery in Africa developing complications while 10% of them die due to postoperative complications [4]. In Sub-Saharan Africa (SSA), laparotomies carry a two-to three-fold increased mortality rate compared to high-income countries [4, 5]. At a national referral hospital in Uganda, 52.3% of patients who had undergone laparotomy developed postoperative complications with an associated mortality rate of 14.5% [6]. Despite current advances in surgical skills and management protocols for patients with planned laparotomy, the rate of developing complications and mortality is still high.

Effective perioperative management of patients undergoing laparotomies is crucial towards the reduction of postoperative morbidity and mortality which could be achieved through the use of objective risk scoring systems [7]. The use of “gut feeling” a subjective predictor of complications or death has been proposed as one of the causes of inadequate postoperative management [8]. Risk stratification is essential in the selection of patients at high risk of postoperative complications for aggressive treatment or the instigation of specific interventions in the immediate postoperative period to mitigate the development of complications and prevent death [9, 10]. Several risk scoring systems which are composed of both objective and subjective variables to predict postoperative morbidity and mortality [11, 12] have been proposed for use and are dependant on when the assessment of the patient due for surgical procedures is done [13].

The Surgical Apgar Score (SAS) is a 10-point score that uses three intraoperative parameters; the lowest heart rate, the lowest mean arterial pressure (MAP) and the estimated blood loss (EBL) during the surgery to predict postoperative complications and death [14]. It is a simple and easy to use tool with a good discriminatory ability to differentiate between those at high and low risk of developing major complications or death within 30 days of surgery [3, 15, 16]. SAS has been validated in other countries [3, 14–17], but its use in LMIC is low. This study will generate more evidence on the predictive performance of SAS in patients undergoing abdominal surgery in LMIC and could increase its adoption in most LMICs. Therefore, this study aimed to determine the performance of SAS in predicting complications and mortality in patients undergoing laparotomy at Mulago National Referral Hospital, Kampala.

## Methods

### Study design

We conducted a prospective observational cohort study from January 2021 to April 2021 and participants were followed up for 30 days.

### Study setting

Mulago National Referral Hospital (MNRH) is a tertiary hospital located approximately 5 km North-East of Kampala Central Business District. It offers specialized care to patients referred from other regions of Uganda and serves as a teaching hospital of the College of Health Sciences, Makerere University. At the surgical outpatient (SOPD), elective cases for laparotomies are booked while the Accident and Emergency (A&E), handles patients requiring urgent intervention who are later transferred to the surgical wards or intensive care unit (ICU). On average, over 40 emergency laparotomies and ten elective laparotomies are conducted at MNRH monthly.

### Study population

Participants who were more than 18 years old that had been admitted and scheduled for either emergency or elective laparotomy at MNRH were recruited. Participants who had polytrauma, metastatic malignancies or those who had undergone major surgical procedures on other body regions including re-laparotomy within 30 days from the time of the first laparotomy were excluded.

#### Sample size estimation

We consecutively recruited 156 participants. Using the sample size formula for comparing two proportions, studies assessing the sensitivity and/or specificity of a single test tool [18] and based on findings from a study done in Kenya [19] and Turkey [20], sample sizes of 365 and 141 participants were calculated, respectively. Due to the small target population, we used a new sample size estimation formula, S = (N)/(1 + N/K) where N is the calculated sample size, K is the maximum population available, and a finite correction factor, K = 200. We considered a 20% loss to follow up and the final sample size of 156 was determined.

### Study procedure

The research assistants introduced the study to prospective participants or their next of kin. For participants who were hemodynamically stable and not in discomfort, the informed consent was obtained prior to the surgery and the research assistant administered an interviewer- guided questionnaire. Participants who were critically ill or those in severe discomfort or pain were consented and recruited after administering the intervention (laparotomy or analgesia).

### Study variables collected

We collected data on the socio-demographic characteristics such as age, and clinical data such as presence of co-morbidities, nature of operation (elective or emergency), duration of surgery, cadre of surgeon (surgical resident or specialist), intra-operative diagnosis (pathology or condition identified upon intraperitoneal access). For admission to ICU, all participants scheduled to have laparotomies were assessed for the need of admission to ICU before, during and after the surgeries. To plan for ICU admission, we assessed for the need of mechanical ventilation (advanced respiratory support) for more than 24 h and/or the need to support two or more failing organ systems in the pre- or postoperative period and/ or met the definition for Clavien class IV (requiring readmission to the intensive care unit (ICU) or considered life-threatening) [21]. This assessment was done by the lead surgeon and/or anesthesia provider. Those who met the criteria, were either admitted or considered for ICU admission depending on the availability of ICU bed space.

#### SAS variables

We collected intraoperative parameters of SAS, but no pre-operative parameters were collected. Heart rate and Mean Arterial Pressure (MAP) were obtained from the anaesthesia case logs either electronically on the patients’ monitor or from the patient’s anaesthesia chart after the operation (after skin incision closure). If MAP was not directly recorded, it was calculated from intraoperative recordings of systolic blood pressure (SBP) and diastolic blood pressure (DBP) using the equation: MAP = [SBP + (2 × DBP)]/3. Estimated Blood Loss (EBL) was calculated after the summation of the amount estimated based on the gauze visual analogue (pictorial materials were available in theatre) [22] by the surgeon and/or anaesthetist/anaesthesiologist, the amount of blood in the suction container and blood spillage. The amount of blood in the suction container was determined at the end of surgery after estimation of the peritoneal contamination fluid (gastric, bowel, and other fluids) and normal saline used in lavage. Blood spillage on the theatre floor was determined by visual estimation by the surgeon. The pictorial material showing different estimated amounts of blood absorbed by the gauze or mop was developed by getting the dry weight of the gauze or mop and then later impregnating it with several different known amounts of blood and getting their weight again. The difference was the estimated amount of blood (1 g of blood measured equals 1 ml). SAS (Table 1) was calculated by summing the point scores of the lowest heart rate, lowest MAP and EBL [14]. The SAS was used to stratify the participants into three categories: high score (SAS 0–4), medium score (SAS 5–7), and low score (SAS 8–10).

**Table 1:** Surgical Apgar score

Parameter	0 point	1 point	2 points	3 points	4 points
Estimated blood loss (ml)	> 1000	601–1000	101–600	≤ 100	–
Lowest MAP (mmHg)	< 40	40–54	55–69	≥ 70	–
Lowest heart rate (beats/min)	> 85^a^	76–85	66–75	56–65	≤ 55^a^

^a^Pathological bradyarrhythmia, sinus arrest, atrioventricular block or dissociation, junction or ventricular escape rhythms and asystole receive 0 points for lowest heart rate

We recorded in-hospital postoperative major complications and mortality based on patient’s outcome in the operating room, recovery room, A&E unit and during their admission in the general surgery ward and ICU. For ease of follow-up, telephone contacts of either the participant or next of kin were recorded in a separate form which was kept by the principal investigator, or a research assistant designated by the principal investigator. We followed up participants on postoperative day 1, day 3, day 5, and every other day until discharge, death or 30^th^ postoperative day. During the follow-up visits, we reviewed clinical notes and recorded patient reported symptoms to identify any post-operative complications or death.

### Outcomes

The outcomes of our study were development of major post operative complications or death. Major complications assessments were based on clinical definitions and were defined based on the American College of Surgeons National Surgical Quality Improvement Program (ACS-NSQIP) [23]. These included: Pneumonia: Chest radiographs with new or progressive and persistent infiltrates, or consolidation, or cavitation, and at least one of the following: (i) fever (> 38 °C) with no other recognized cause, or (ii) leucopenia (< 4000 white blood cells/mm3) or leukocytosis (> 12,000 white blood cells/mm^3^), (iii) new onset of purulent sputum or change in the character of sputum, or increased respiratory secretions, or increased suctioning requirements, (iv) new onset or worsening cough, or dyspnea, or tachypnoea, with rales or bronchial breath sounds.

#### Deep surgical site infection (deep)

AN infection within 30 days after surgery if no surgical implant is left in place which involves deep soft tissues of the surgical incision (for example, fascial and muscle layers) and a patient had at least one of the following: (a) Purulent drainage from the deep incision. (b) deep incision that spontaneously dehisced or was deliberately opened by a surgeon or attending physician and was culture-positive or no cultures were taken, and the patient had at least one of the following symptoms: fever (> 38 °C); localized pain or tenderness, (c) an abscess or other evidence of infection involving the deep incision that is detected on gross anatomical exam, or imaging test.

#### Surgical site infection (organ/space)

Infection involves any part of the body deeper than the fascial/muscle layers, that was opened or manipulated during the operative procedure and patient had at least one of the following: (a) Purulent drainage from the drain that was placed into the organ/space through a stab wound into the organ/space, (b) Organism identified from an aseptically obtained fluid or tissue in the organ/space by culture or non-culture based microbiologic testing method which was performed for purpose of clinical diagnosis or treatment, (c) An abscess or other evidence of infection involving the organ/space that is found on direct examination, during reoperation, or by radiologic examination, or d) diagnosis of an organ/space surgical site infection by a surgeon or attending physician.

#### Wound dehiscence

Superficial or deep wound breakdown.

#### Acute kidney injury

Increase in serum creatinine level 2.0 to 3.0-fold or serum creatinine level greater or equal to 4 mg/dl (≥ 354 μmol/l) with an acute increase of > 0.5 mg/dl (> 44 μmol/l) or the initiation of renal replacement therapy, or urine output < 0.5 ml/kg/h for 12 h or anuria for 12 h. Stage 2 and 3 Acute kidney injury as defined by Acute Kidney Injury Working Group of KDIGO (kidney disease: Improving Global Outcomes) [24].

#### Cardiac arrest

The cessation of cardiac mechanical activity, as confirmed by the absence of signs of circulation (absence of a palpable central pulse or bradycardia with less than 60 beats per minute (bpm) with poor perfusion requiring external cardiac compressions and assisted ventilation), unresponsiveness and no respiratory effort.

#### Anastomotic leak

Discharge of bowel contents via the drain, wound or abnormal orifice.

Unplanned intubation: requiring placement of an endotracheal tube secondary to the onset of respiratory or cardiac failure as evidenced by severe respiratory distress, hypoxia, hypercarbia, or respiratory acidosis within 30 days of the operation (definition by ACS-NSQIP database). For patients who were intubated for surgery, any intubation after prior intubation was considered unplanned intubation even.

#### Septic shock

Sepsis-induced persistent hypotension (systolic blood pressure < 90 mmHg and diastolic < 60 mmHg) despite adequate fluid resuscitation along with the presence of perfusion abnormalities that may include, but are not limited to, lactic acidosis, oliguria, or an acute alteration in mental status [25].

Post operative complications which met the definition for Clavien class III complications (requiring surgical, endoscopic, or radiologic intervention) and class IV (requiring readmission to the intensive care unit [ICU] or considered life-threatening) were categorized as major complications [21]. Multiple complications in a single patient were graded and recorded separately. Patients’ outcomes (alive or dead, major complication or no major complication) were the point of reference against which SAS was compared.

#### Post-operative mortality

Data on deaths of participants post-operatively was generated from the medical certificate of death.

### Quality assurance and control

Questionnaires were pre-tested to capture all the data required to answer the research objectives. All research assistants were trained prior to the commencement of the study in Human Subjects Protection short course, the different research procedure, and the full research protocol. All entered data was validated using data cleaning codes and programs and questionnaires were cross checked for completeness.

### Analysis

Data was entered into EPI-DATA 4.2 and exported to STATA version 16 for analysis. Baseline characteristics and continuous variables are summarized using means and standard deviations or medians and interquartile ranges for normally distributed and skewed data, respectively. Categorical variables were summarized using proportions and percentages where appropriate. Tables, bar graphs and pie chart where appropriate are used to present results. The 30-day post-operative survival rates were calculated using the Kaplan–Meier method. The chi-square test was used to determine the association between major complications and the independent variables (SAS categories (0–4, 5–7, 8–10), age, sex, nature of the operation, the cadre of the surgeon, needing ICU, intraoperative diagnosis, and duration of operation). In addition, Chi-square was used to determine the association between being alive or dead and the SAS. Variable with a cut-off p-value less than or equal to 0.20 at bivariate analysis and those clinically known to be associated with major complication were subjected to multivariate logistic regression adjusting for potential confounders. A p-value of 0.05 or less was considered statistically significant. To test the surgical Apgar score’s discriminatory ability for complications, the area under the receiver operating characteristic (ROC) curves were generated. The patient’s outcome (alive or dead, major complication, or no major complication) were the references against which SAS was compared. The point estimate on the ROC curves whose sensitivity and specificity had the maximal Youden’s index ([Sensitivity + specificity] − 1) was the optimal cut-off and its corresponding sensitivity, specificity, and the area under curve (AUC) was reported. The same was done for mortality.

## Results

### Participant demographics

**Fig. 1:**
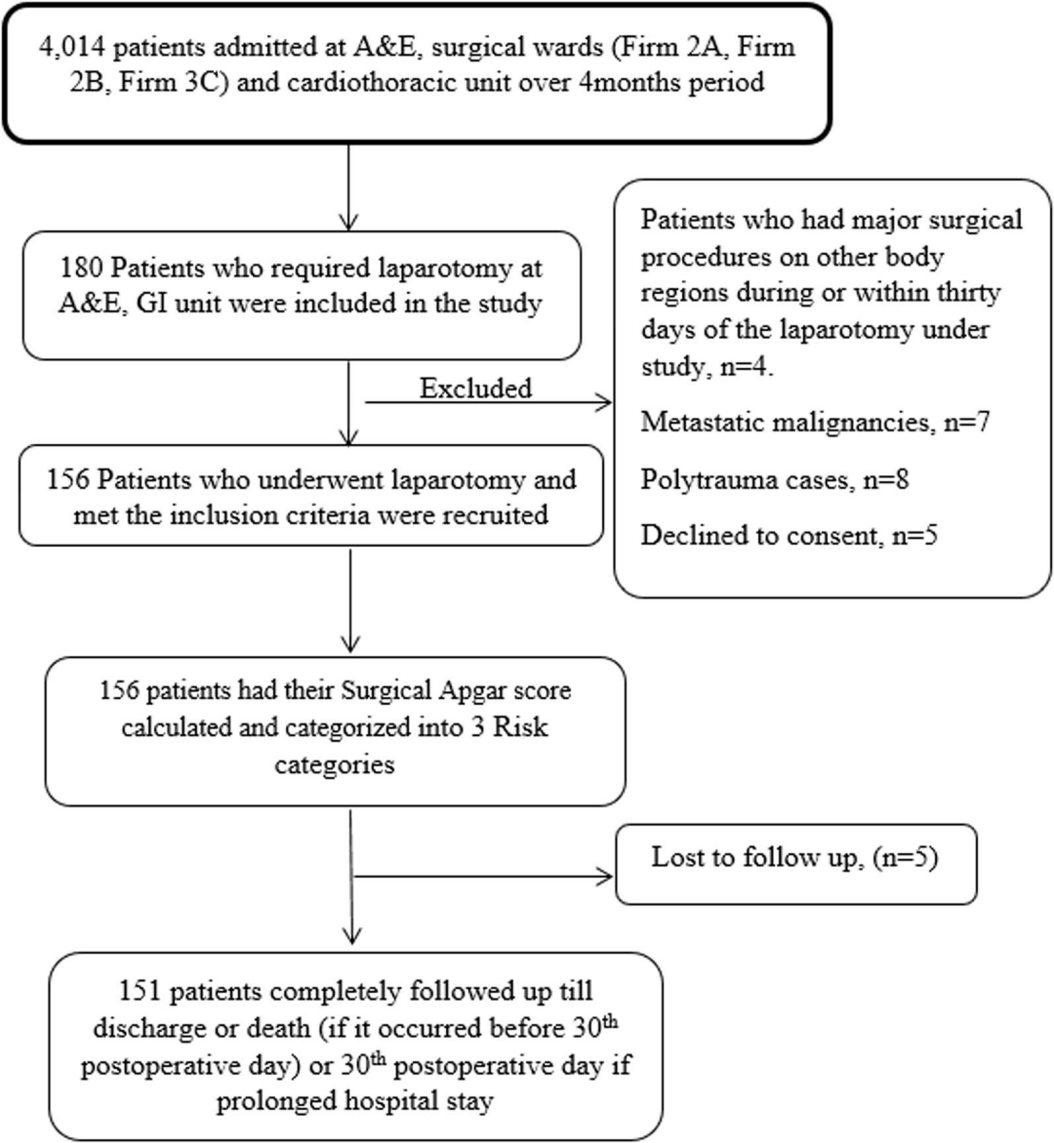
Study flow chart

We recruited 156 participants into the cohort but five were lost to follow-up (Fig. 1). One hundred fifty-one participants were included in the final analysis. Of the 151 participants, 103 (68.2%) were male, and the mean age was 40.6 ± 15 years. Seventeen (11.3%) had co-morbidities with hypertension the most common at seven (41.1%).

**Fig. 2:**
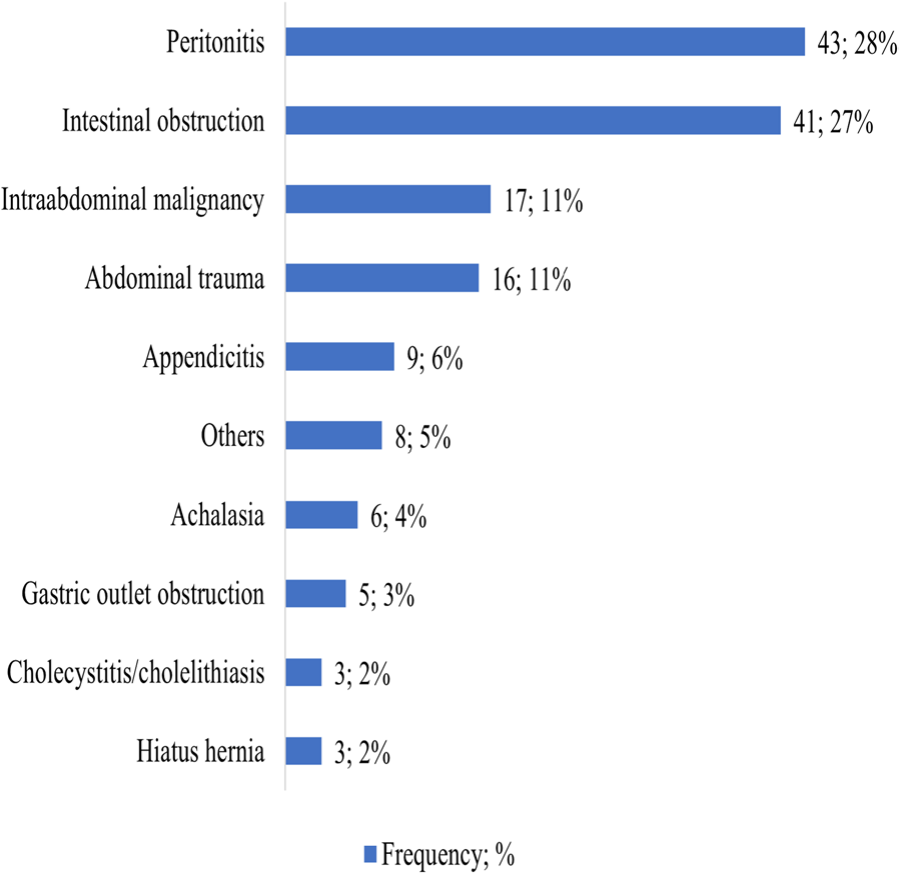
Distribution of clinical indications among patients underwent laparotomy at Mulago National Referral Hospital

Regarding clinical characteristics, the majority of the participants underwent emergency laparotomies (108, 71.5%). General Surgery residents conducted most of the laparotomies, 107 (70.9%), with emergency laparotomies accounting for 103 (96.3%). The most common reported indication for laparotomies was peritonitis at 43 (28.5%) followed by intestinal obstruction at 41(27%) (Fig. 2) while gastrointestinal perforation at 47 (31.1%) was the most reported intraoperative diagnosis. Other patient characteristics are summarized in Table 2. In terms of clinical outcomes, 37 (24.2%) of the participants developed major complications with 20 (13.2%) having 3 or more major complications and 16 (10.6%) participants died following surgery (Table 3). The median duration of developing post operative cardiac arrest was 0.5 (IQR: 0–1) which was the shortest while participants took a median duration of 6 days post operative to develop pneumonia (IQR, 4–10), wound dehiscence (IQR, 5–6), and anastomotic leak (IQR, 5–8). About 14 patients (9.3%) were re-operated, 7 of whom were due to anastomotic leaks.

**Table 2:** Participant's characteristics

Variable	Frequency	%
Age		
Mean, SD	40.61(± 15.74)	
Sex		
Male	103	68.2
Female	48	31.8
Presence of comorbidity		
Yes	17	11.3
No	134	88.7
Specify comorbidities (n = 17)		
Hypertension	7	4.6
HIV	5	3.3
Diabetes mellitus only	2	1.3
Hypertension + diabetes	2	1.3
Liver cirrhosis	1	0.7
Clinical characteristics		
Category of operation		
Emergency	108	71.5
Elective	43	28.5
Cadre of primary surgeon		
Resident	107	70.9
Emergency	103	96.3
Elective	4	3.7
Specialist	44	29.1
Emergency	5	11.4
Elective	39	88.6
Intra-operative diagnosis		
Gastrointestinal perforation	47	31.1
Gut obstruction	41	27.2
Intraabdominal malignancy	14	9.3
Inflammatory disorder	18	11.9
Others	31	20.5
Duration of surgery (median minutes/IQR)	125	90–180
≤ 120 min	59	39.1
> 120 min	75	49.7

**Table 3:** Outcomes in patients undergoing laparotomy ant Mulago National Referral Hospital

Variables		
Occurrence of major complications		
No	114	75.5
Yes	37	24.5
Number of major complications		
1–2 major complications	17	11.3
3 or more major complications	20	13.2
Mortality		
Survivors	135	89.4
Non-survivors	16	10.6
Individual major complications		
Pneumonia	7	4.6
Post-operative day (median. IQR)	6	4–10
Wound dehiscence	13	8.6
Post-operative day (median. IQR)	6	5–6
Deep or organ-space SSI	26	17.2
Post-operative day (median. IQR)	5	5–7
Reoperation	14	9.3
Post-operative day (median. IQR)	6	6–7
Anastomotic leak	10	6.6
Post-operative day (median. IQR)	6	5–8
Cardiac arrest	7	4.6
Post-operative day (median. IQR)	0.5	0–1
Acute kidney injury	4	2.6
Post-operative day (median. IQR)	4.5	2—6.5
Septic shock	4	2.6
Intubation	4	2.6
Post-operative day (median. IQR)	5	0–10
Admission to Intensive Care Unit (ICU)		
Planned Admission to ICU	14	9.3
Admitted to ICU	5	35.7
Not admitted to ICU	9	64.3

### Post-surgery survival

**Fig. 3:**
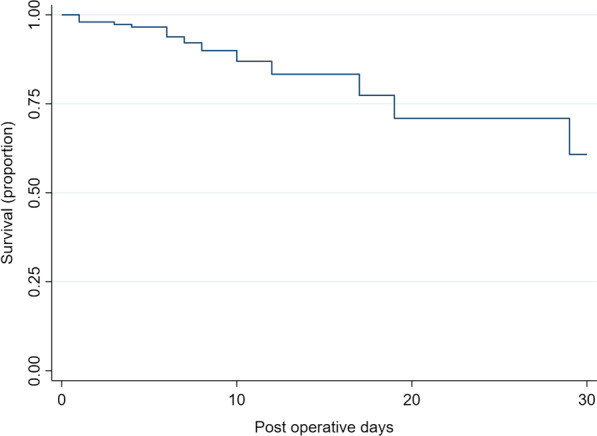
The Kaplan-Meir survival estimates during the period of follow up

Patients were followed up for 30 days after surgery. Figure 3 shows the Kaplan-Meir survival estimates during the period of follow up. Survival at day 1 was 98.7% (IQR: 94.8–99.7%), 96.7% (92.2–98.6%) at day 3, 91.7% (84.6–95.6%) at day 7, 85.6% (76.6–91.4%) at day 14, 83.1% (73.5–89.4%) at day 21 and 81.4% (71.4–88.2%) at day 30.

### Surgical Apgar score (SAS): score category distribution, components, and diagnostic accuracy

In our study, 99 (65.6%) of the participants had a medium SAS (Table 4). The median estimated blood loss was 127 mls (Interquartile range (IQR), 84–124) while the median lowest heart rate was 82 bpm (IQR 70–100). The median lowest MAP was 70 mmHg, (IQR 63–78).

**Table 4:** Distribution of SAS parameters

Variable	Frequency	%
SAS (median, IQR)	6	IQR (5–7)
SAS risk categorization		
Low risk (8–10)	20	13.2%
Medium (5–7)	99	65.6%
High risk (0–4)	32	21.2
Estimated blood loss in mls (median, IQR)	127	84–127
≤ 100	58	38.41
101–600	69	45.7
601–1000	12	7.95
> 1000	12	7.95
Lowest heart rate in beats per minute (median, IQR)	82	70–100
≤ 55	6	3.97
56–65	19	12.58
66–75	29	19.21
76–85	34	22.52
> 85	63	41.72
Lowest mean arterial pressure (median, IQR)	70	63–78
≥ 70	78	51.66
55–69	58	38.41
40–54	15	9.93

**Fig. 4:**
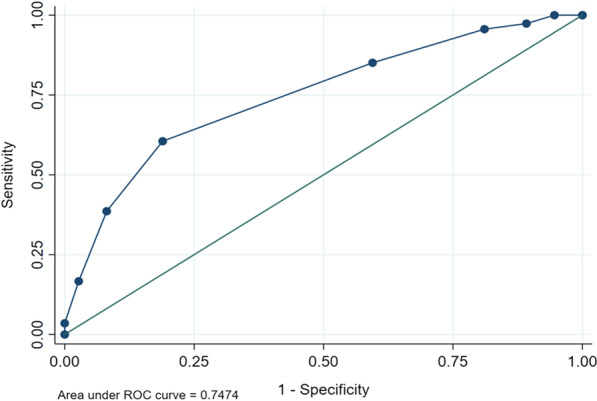
Receiver operating characteristic (ROC) curves for surgical Apgar score and in-hospital major complications

**Fig. 5:**
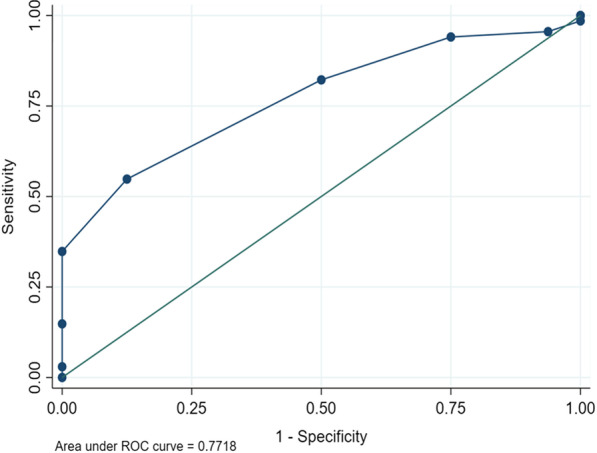
Receiver operating characteristic (ROC) curves for surgical Apgar score and post operative mortality

SAS had fair discriminatory ability with the AUC for in-hospital major complications (Fig. 4) at 0.75 (95% CI, 0.68–0.87) while that of mortality (Fig. 5) at 0.77 (95% CI, 0.66–0.83). From the ROC curve analysis, SAS ≤ 6 had the highest Youden’s index of 0.42 hence the optimal cut-off. A SAS ≤ 6 had a sensitivity of 60.5% and specificity of 81.1% for detecting complications for patients undergoing laparotomy. For mortality, a SAS ≤ 6 had a sensitivity of 54.8% and specificity of 87.5% (Youden’s index of 0.42) for mortality in patients undergoing laparotomy.

### Factors associated with major postoperative complications and mortality

Based on bivariate analysis (Table 5), the crude relative risk of participants developing major complications in the high SAS category was 16.8 (95% CI, (2.0–140.7), P = 0.009) compared to the low SAS category. Participants in the high SAS category were 3.8 times more likely to die compared to those in the medium SAS category (95% CI, (1.3–11.1), P = 0.015). Emergency laparotomies were 9.8 times (CRR, 95% CI, 2.2–43.0), p < 0.05) compared to the elective cases. Participants who required ICU admission were 10.2 times (95% CI (3.0–35.0), p < 0.05) and 18.2 times (95% CI (5.2–65.3), p < 0.001) as likely to develop complications and to suffer death, respectively as those who did not require ICU admission.

**Table 5:** Factors associated with major complications and mortality

Outcome variables	Major complications				Mortality			
	Crude RR (95%CI)	p- value	Adjusted RR (95% CI)	p-value	Crude RR (95% CI)	p-value	Adjusted RR (95% CI)	p-value
SAS	0.5 (0.4–0.7)	< 0.001	0.5 (0.4–0.7)	< 0.001	0.6 (0.4–0.8)	0.001	0.6 (0.4–0.8)	0.001
SAS category								
Low score (n = 21)	1.0		1.0		NA		N/A	
Medium score (n = 98)	5.1 (0.6–40.4)	0.122	3.5 (0.4–29.3)	0.243	1.0		1.0	
High score (n = 32)	16.8 (2.0–140.7)	0.009	18.4 (1.9–177.0)	0.012	3.8 (1.3–11.1)	0.015	3.9 (1.01–15.3)	0.048
Age (years)								
65 or more	1.0		1.0		1.0		1.0	
18–64	1.9 (0.4–8.8)	0.430	2.4 (0.3–21.3)	0.436	1.7(0.3–8.6)	0.532	2.9 (0.3–29.2)	0.364
Sex								
Female	1.0				1.0			
Male	1.6 (0.7–3.8)	0.264			0.7 (0.2–2.1)	0.520		
Nature of operation								
Elective	1.0		1.0		1.0		1.0	
Emergency	9.8 (2.2–43.0)	0.002	19.5(1.1- 350.5)	0.044	6.3 (0.8 – 49.6)	0.081	2.1 (0.03–147.3)	0.736
Cadre of surgeon								
Specialist	1.0		1.0		1.0		1.0	
Residents	6.4 (1.8–22.0)	0.003	1.2 (0.1–14.2)	0.872	6.6 (0.8 – 51.6)	0.074	7.1 (0.1–524.2)	0.370
Duration of surgery								
≤ 120 min	1.0				1.0			
> 120 min	1.1 (0.5–2.4)	0.730			1.3 (0.4 – 3.9)	0.606		
Intraoperative diagnosis								
Intestinal obstruction	1.0				N/A			
GI perforation	7.9 (2.8–22.3)	< 0.001						
Inflammatory disorder	1.6 (0.3–7.4)	0.555						
Others	0.2 (0.02–1.7)	0.140						
Need ICU^a^								
No	1.0		1.0		1.0		1.0	
Yes	10.2 (3.0–35.0)	< 0.001	16.3 (2.8–94.6)	0.002	18.2 (5.2 – 65.3)	< 0.001	23.9 (4.9–115.9)	< 0.001

^a^*ICU* Intensive Care unit

On multivariate analysis (Table 5), SAS was an independent risk factor for complications and mortality post-operatively. Patients in the high-SAS category had a high likelihood of developing major complication (ARR, 95% CI, (18.4 (1.9–177.0), p = 0.012), and dying (ARR, 95% CI, 3.9 (1.01–15.26), p = 0.048) compared to those in the medium SAS category. Emergency laparotomies were associated with complications post operatively (AAR, 95% CI, 19.5 (1.1–350.5), p = 0.044). In addition, the need for ICU admission was associated with post operative complications (ARR, 95% CI, 16.3 (2.8–94.6), p < 0.05) and mortality (ARR, 95% CI, 23.9 (4.9–115.9), p < 0.001). However, there was no statistical significance between cadre of primary surgeon and post operative complications and mortality.

## Discussion

In our cohort study, we investigated the performance of SAS in predicting postoperative major complications and mortality among participants who had undergone laparotomies at MNRH.

The observed in-hospital mortality rate in our study was 10.6%. This is consistent with prior studies in resource-limited areas which reported a mortality rate ranging between 5.5% and 22.4% [4, 6, 17, 19, 26, 27]. Compared to findings from a global survey [28], we had a higher mortality rate in our cohort. This may be attributed to the fact that we had mainly emergency cases who were not adequately optimized and majority had delays in making diagnosis and surgical intervention. In our study, the overall in-hospital complication rate was 24.5%. This is in agreement with findings from a study in northern Uganda [29] and Rwanda [17] where the complication rate was 24.2% and 29%, respectively. In our setting, patients are delayed or misdiagnosed at other lower healthcare facilities which may cause their clinical deterioration preoperatively, intraoperatively, and post operatively.

We found that the SAS had a fair discriminatory ability for in-hospital complications and mortality with an AUC of 0.75 (95% CI, 0.64–0.82) and 0.77 (95% CI, 0.61–86), respectively. Our findings were in agreement with those from a study conducted in Rwanda which had an AUC of 0.79 for postoperative in-hospital mortality and 0.7 for major complications [17]. Similarly, in a multi country pilot study, the AUC of SAS was 0.70 and 0.77 for prediction of any complication and mortality, respectively [30] while among 1,441 patients undergoing general and vascular surgical procedures, SAS achieved a C statistic of 0.73 for predicting major complications and 0.81 for predicting deaths [15]. In another study conducted among patients undergoing emergency abdominal surgery, SAS had a relatively weak discriminatory power with an AUC of 0.63 [31] which was lower than AUC in our study. The low AUC could be due to the perioperative patient optimization which could have affected the scores. SAS had a low sensitivity in predicting the development of complications and mortality post operatively but had a high specificity in predicting the development of complications and mortality among participants who had had laparotomies. This agrees with findings from a retrospective study done in the Caribbean [20]. Due to its predictive ability, SAS provides a potential platform to identify patients at risk of mortality and morbidity so that aggressive management plans can be instituted. Form our study, patients with a SAS of ≤ 6 should have their post-operative management plan re-evaluated and revised to reduce the risk of morbidity and mortality.

In our study, high SAS category, emergency laparotomies, and the need for ICU were associated with complications post operatively while high SAS category as well as needing ICU were associated with mortality. A pilot study of SAS in general and vascular patients, patients in the high SAS category were 16 times at greater risk of experiencing a major complication compared to those in the low and medium SAS category [14]. In addition, Regenbogen and colleagues found that participants within the high SAS category were 112.0 times more likely to die (95% CI, (15.3–819.7); p < 0.001) within 30 days compared to the those with medium and low SAS categories [32]. The high risk of developing complications and mortality post operatively could be attributed to the high number of surgeries conducted by residents who may have committed errors leading to intraoperative bleeding. Additionally, majority of our participants scheduled for emergency laparotomy had the surgery more than 72 h after initial symptom onset which could have affected their intraoperative and post operative conditions. The delayed laparotomy of participants who were at an irreversible physiological deterioration stage made them unsalvageable even with the appropriate treatment or intervention. Like in Rwanda, emergency status were associated with significantly increased risk of postoperative major complications and death when compared to elective surgeries [17]. The need for ICU admission was associated with complications and mortality post operatively. This may be due to the unstable preoperative status of the participants which could have negatively affected the intraoperative and post operative states of the participants hence the high risk.

### Study limitations

Different gauze material weight and mixture of blood with peritoneal contaminants (bowel contents, pus, or fluid) in the suction container may have resulted in over estimation of blood loss while underestimation of blood loss may have resulted from blood absorbed by the linen and spillage on the floor. This affected the objective total blood loss estimated. However, the wide categorization of blood loss used allows for a reasonable accurate estimation since it is easily within the observers’ range of precision.

Perioperative haemodynamic (blood pressure, pulse rate and mean arterial pressure) were affected by anaesthetic drugs, depth of anaesthesia and interventions, which could have altered the physiological status of participants. Additionally, preoperative fluid resuscitation state of the patient could have affected intraoperative hemodynamic state. This could have affected the computation of the SAS leading to misclassification of patients and may have contribute to a high or low complication and/or mortality rate in the different SAS categories. In our study, we did not collect data on the pre-operative status of participants and future studies should explore how pre-operative status affects the predictive ability of SAS.

Overall complication and mortality may have been underestimated due to premature discharge of participants and the study examining only inpatient complications or mortality. We were unable to assess for neurological complications and future studies should explore the incidence/prevalence of neurological complications among post-operative elderly patients. In addition, SAS has been shown to predict ICU admission in high risk abdominal surgeries [33], more studies could explore this outcome in LMICs where available surgical resources differ. Coming from a single tertiary centre like Mulago hospital, our results lack generalizability to Uganda as a whole.

## Conclusion

Low SAS (≤ 6) is associated with increased risk of developing in-hospital major complications and/or death following laparotomy at Mulago Regional Referral Hospital.

SAS can adequately predict, or risk stratify patients undergoing laparotomy in a low resourced Centre (MNRH) at higher-than-average risk of developing in-hospital postoperative major complications and/or dying.

SAS has a high specificity with an overall fair discriminatory ability for predicting those at high or low risk of developing in-hospital major complications and/or death following laparotomy in a low resourced tertiary hospital in Uganda.

### Recommendations

SAS should be adopted by the department of surgery Mulago National Referral hospital and used to assist the surgical team in predicting or stratifying patients at high or low risk of developing postoperative in-hospital major complications and/or dying.

Multicentered studies to evaluate the performance of SAS at different level of care in Uganda should be done before generalization of these result to the country.

## Data Availability

Data supporting the results reported in this article has been availed in Fig share repository, https://doi.org/10.6084/m9.figshare.21209558.v2.

## Abbreviations

A&E: Accident and emergency
ACS: American College of Surgeons
EBL: Estimated blood loss
HIC: High income countries
ICU: Intensive care unit
LMICs: Low- and middle-income countries
MAP: Mean arterial pressure
MNRH: Mulago National Referral Hospital
NSQIP: National Surgical Quality Improvement Program
SAS: Surgical Apgar score
SOPD: Surgical outpatient department
WHO: World Health Organization

## References

[1] DeathCollaborators GCo. Global, regional, and national age-sex specific mortality for 264 causes of death, 1980–2016: a systematic analysis for the Global Burden of Disease Study 2016. The Lancet. 2017;390(10100):1151–210. 10.1016/S0140-6736(17)32152-9 PMC5605883 28919116

[2] Nepogodiev D, Martin J, Biccard B, Makupe A, Bhangu A (2019). Surgery NIfHRGHRUoG. Global burden of postoperative death. Lancet (London, England)..

[3] Haynes AB, Regenbogen SE, Weiser TG, Lipsitz SR, Berry WR, Gawande AA (2009). Surgical outcome measurement for a global patient population: validation of the Surgical Apgar Score in eight countries. J Am Coll Surg.

[4] Biccard B, Madiba T, Kluyts H, Munlemvo D, Madzimbamuto F, Basenero A, African Surgical Outcomes Study (ASOS) investigators (2018). Perioperative patient outcomes in the African Surgical Outcomes Study: a 7-day prospective observational cohort study. The Lancet.

[5] Collaborative G (2016). Mortality of emergency abdominal surgery in high-, middle- and low-income countries. Br J Surg.

[6] Kitara D, Kakande I, Mugisa J (2010). The postoperative complications prediction in Mulago Hospital using POSSUM scoring system. East Central Afr J Surg.

[7] Sehgal S, Ravishankar N, Raghupathi DS, Kotekar N (2019). Can the surgical Apgar score predict morbidity and mortality in general surgery?. International Surgery Journal.

[8] Dilaver N, Gwilym B, Preece R, Twine CP, Bosanquet DCJBO (2020). Systematic review and narrative synthesis of surgeons' perception of postoperative outcomes and risk. BJS Open.

[9] Hussain A, Mahmood F, Teng C, Jafferbhoy S, Luke D, Tsiamis A (2012). Patient outcome of emergency laparotomy improved with increasing "number of surgeons on-call" in a university hospital: audit loop. Annal Med Surg.

[10] Huddart S, Peden C, Swart M, McCormick B, Dickinson M, Mohammed MA (2015). Use of a pathway quality improvement care bundle to reduce mortality after emergency laparotomy. J Br Surg.

[11] Knaus WA, Draper EA, Wagner DP, Zimmerman JE (1985). APACHE II: a severity of disease classification system. Crit Care Med.

[12] Copeland GPJAOS (2002). The POSSUM system of surgical audit. Arch Surg.

[13] Wolters U, Wolf T, Stützer H, Schröder T (1996). ASA classification and perioperative variables as predictors of postoperative outcome. Br J Anaesth.

[14] Gawande AA, Kwaan MR, Regenbogen SE, Lipsitz SA, Zinner MJ (2007). An Apgar score for surgery. J Am Coll Surg.

[15] Regenbogen SE, Ehrenfeld JM, Lipsitz SR, Greenberg CC, Hutter MM, Gawande AA (2009). Utility of the surgical Apgar Score: validation in 4119 patients. Arch Surg.

[16] Regenbogen SE, Lancaster RT, Lipsitz SR, Greenberg CC, Hutter MM, Gawande AA (2008). Does the surgical Apgar Score measure intraoperative performance?. Ann Surg.

[17] Ngarambe C, Smart BJ, Nagarajan N, Rickard J (2017). Validation of the surgical apgar score after laparotomy at a tertiary referral hospital in Rwanda. World J Surg.

[18] Hajian-Tilaki K (2014). Sample size estimation in diagnostic test studies of biomedical informatics. J BioMed Informat.

[19] Dullo M. Surgical apgar score: applicability in patients undergoing laparatomy at Kenyatta National Hospital: University of Nairobi; 2013.

[20] Singh K, Hariharan S (2019). Detecting major complications and death after emergency abdominal surgery using the surgical Apgar score: a retrospective analysis in a Caribbean setting. Turkish J Anaesthesiol Reanim.

[21] Dindo D, Demartines N, Clavien P-A (2004). Classification of surgical complications. Ann Surg.

[22] Algadiem EA, Aleisa AA, Alsubaie HI, Buhlaiqah NR, Algadeeb JB, Alsneini HA. Blood loss estimation using gauze visual analogue. Trauma Monthly. 2016;21(2). 10.5812/traumamon.34131 PMC5003499 27626017

[23] Khuri SF, Daley J, Henderson W, Barbour G, Lowry P, Irvin G (1995). The National Veterans Administration Surgical Risk Study: risk adjustment for the comparative assessment of the quality of surgical care. J Am Coll Surg.

[24] KDIGO. KDIGO 2012 Clinical Practice Guideline for the Evaluation and Management of Chronic Kidney Disease. J Int Soc Nephrol. 2012;3(1). 10.1038/ki.2013.243 23989362

[25] Gül F, Arslantaş MK, Cinel İ, Kumar A (2017). Changing definitions of sepsis. Turk J Anaesthesiol Reanim.

[26] Hewitt-Smith A, Bulamba F, Olupot C, Musana F, Ochieng J, Lipnick M (2018). Surgical outcomes in eastern Uganda: a one-year cohort study. South Afr J Anaesthesia Analgesia.

[27] Sincavage J, Msosa VJ, Katete C, Purcell LN, Charles A (2021). Postoperative complications and risk of mortality after laparotomy in a resource-limited setting. J Surg Res.

[28] Collaborative G (2018). Surgical site infection after gastrointestinal surgery in high-income, middle-income, and low-income countries: a prospective, international, multicentre cohort study. Lancet Infect Dis.

[29] Okeny PK, Hwang TG, Ogwang DM. Acute bowel obstruction in a rural hospital in Northern in Northern Uganda. East Central Afr J Surg. 2011;16(1).

[30] Haynes AB, Regenbogen SE, Weiser TG, Lipsitz SR, Dziekan G, Berry WR (2011). Surgical outcome measurement for a global patient population: validation of the Surgical Apgar Score in 8 countries. Surgery.

[31] Cihoric M, Toft Tengberg L, Bay-Nielsen M, Bang FN (2016). Prediction of outcome after emergency high-risk intra-abdominal surgery using the surgical Apgar Score. Anesth Analg.

[32] Regenbogen SE, Ehrenfeld JM, Lipsitz SR, Greenberg CC, Hutter MM, Gawande AA. Utility of the surgical apgar score: validation in 4119 patients. Archives of Surgery (Chicago, Ill: 1960). 2009;144(1):30–6; 10.1001/archsurg.2008.504 19153322

[33] Sobol JB, Wunsch H, Li G (2013). The Surgical Apgar Score is strongly associated with ICU admission after high-risk intra-abdominal surgery. Anesth Analg.

